# Supplementary material for: Factors associated with insufficient weight gain among Mexican pregnant women with HIV infection receiving antiretroviral therapy
Source: PLoS One. 2020 May 22;15(5):e0233487. doi: 10.1371/journal.pone.0233487 (PMC7244146; doi:10.1371/journal.pone.0233487)
Supplement: Attachment — Submitted filename: PLOS ONE Review.pdf [file pone.0233487.s002.pdf]

PLOS ONE Review: INFLUENCING FACTORS CONCERNING INSUFFICIENT WEIGHT GAIN ON MEXICAN PREGNANT WOMEN WITH HIV AND ANTIRETROVIRAL THERAPY

Overall: This is an important article and should be published. I suggest the employment of an academic editor, as the English presentation of the work is distracting from the results. Although this study focuses on the 112 women receiving ART, I suggest a similar study be accomplished on the general population of Mexican women, so the results of the women receiving ART be placed in appropriate academic context.

Introduction:
"Concerning to a cohort study on HIV seropositive pregnant women who started ART, this analysis found that patients who had a weekly gestational weight gain of less than 100 grams, displayed a higher risk of preterm birth and adverse pregnancy-neonatal outcomes such as low birth weight, concluding that the use of ART is not enough in this kind of population to reduce the risk of these results, because is prevailing a poor nutritional status in spite of ART [12,13]."
--How does this relate to the population of Mexican women that had a weekly gestational weight gain of less than 100 grams and were not HIV seropositive? Answering this question may shift the context of results from the involvement of ART to simply weight gain.

The final paragraph seems to indicate a relationship between the receipt of ART and nutritional status. This linkage is unclear, except in that ART is shown to slow the progress of HIV and therefore slow the deterioration of the patient. However, this is different from linking ART to weight gain and improved nutritional status.

Results:
The statistics stated in the first paragraph are different than those reported in the abstract.

I do not understand the significance of these results: "The percentage of adequacy in energy consumption ($r = .22$), iron ($r = .20$), folic acid($r = .20$) and zinc ($r = .29$), as well as the ingesting in grams of protein ($r = .29$), total fat ($r = .20$), saturated ($r = .28$), monounsaturated ($r = .26$), cholesterol ($r = .20$) and fiber ($r = .20$); they correlated with the socioeconomic level ($p <0.05$)."

This statement seems to indicate the authors completed a broader analysis of Mexican women "However, the mainstream (40.2%) showed an insufficient weight gain or weight loss at the time of the evaluation and only 35.7% had a weight gain according to weight gain recommendations [20]." This information must be made more clear.

Discussion:
The observation surrounding women in unions being at higher risk for insufficient weight gain is important. The authors should expand this and look at other correlative and potentially causal factors.

Based upon the comparison of vitality in Mexican men with HIV compared to that of pregnant Mexican women with ART, it seems like the underlying causal social dynamics could be the subject of an entire other article. It is important and should be publicized.

The final paragraph is exactly right. This work has the potential to make a significant impact.
