# Supplementary material for: Factors associated with insufficient weight gain among Mexican pregnant women with HIV infection receiving antiretroviral therapy
Source: PLoS One. 2020 May 22;15(5):e0233487. doi: 10.1371/journal.pone.0233487 (PMC7244146; doi:10.1371/journal.pone.0233487)
Supplement: Attachment — Submitted filename: Notes.pdf [file pone.0233487.s003.pdf]

For the best experience, open this PDF portfolio in Acrobat X or Adobe Reader X, or later.

Get Adobe Reader Now!
