# Supplementary material for: Factors associated with insufficient weight gain among Mexican pregnant women with HIV infection receiving antiretroviral therapy
Source: PLoS One. 2020 May 22;15(5):e0233487. doi: 10.1371/journal.pone.0233487 (PMC7244146; doi:10.1371/journal.pone.0233487)
Supplement: Attachment — Submitted filename: Response to Reviewers.docx [file pone.0233487.s004.docx]

December 20, 2019

Diana M. Thomas
Academic Editor
PLOS ONE

Dear academic editor:

Thanks, about your suggestions concerning our paper "Influencing factors concerning insufficient weight gain on Mexican pregnant women with HIV and Antiretroviral therapy” to your consideration for publication in PLOS ONE.

Now, we respond to each of the observations made by academic editor:

1. The entire manuscript was edited by native speaker in (scientific) American English (certificate annex)
2. The pregestational weight was calculated as suggested, according the model to estimate the pregestational weight from the weight in pregnancy that considers height, gestational age, current weight, parity and age using a software (https ://www.pbrc.edu/resea rch-and-facul ty/calculator s/prepregnancy/), reference 21 of the manuscript.

Regarding de comments of the reviewer 1:

The wholw manuscript was edited by native speaker in (scientific) American English

It would be basic, indeed, make a comparative analysis about pregnant women with HIV using antiretrovirales versus healthy population to reach other socioclinical perspectives.

Introduction:

Concerning the reviewer’s enquiring, about the specific question: how does a weekly weight gain of less than 100 grams affect Mexican pregnant women without HIV? , we can say that, according with our bibiography and data, most of the recent studies that have evaluated the determinants of weight gain in Mexican pregnant women have focused on excessive weight gain (Zonana-Nacach A. Salud Publica Mex. 2010;52 (3);220-225), even assessing how it affects some biochemical markers (Perichart-Perera. 2017 PLoS ONE 12(7):e0180874). This approach is related to the high prevalence of overweight and obesity in Mexican women of reproductive age, as well as, the high prevalence of excessive weight gain that has been reported in studies in the Mexican population (33 al 38%). The population of our study cannot be compared with this generality since as mentioned, it presented a very high proportion of insufficient weight gain (62.5%) and even a significant percentage of weight loss (18.8). This is a result not only of their clinical condition but also of the condition of vulnerability in which they find themselves, as has been raised in the manuscript.

A sugerencia del revisor, en el último párrafo de la introducción se aclaró que el uso de los antirretrovirales no se relaciona directamente con el estado nutricio sino más bien su deterioro es parte de la progresión de la enfermedad y la presencia de un pobre estado nutricio a pesar del uso de antirretrovirales puede ser resultado de otros factores.

We agree with your insight stated at the last paragraph of the introduction. We clarified that the use of antiretrovirals is not directly related to nutritional status, but rather, the deterioration is part of the progression of the disease and the presence of poor nutritional status despite the use of antiretrovirals may result from other factors.

Results:

The inconsistency, concerning the results in the summary quoted in the first paragraph was corrected.

The correlation of energy consumption and various nutrients with socio-economic level was considered important to mention, to show how the socio-economic condition affects the consumption not only of energy but also of nutrients, as a result of consumption patterns of low-income populations (lines 260-263).

The description of weight gain in the study population was expanded, the information was presented more clearly (lines 163-168)

Discussion:

The discussion surrounding women in unions being at higher risk for insufficient weight gain was more expand (lines 227-235).

We agree with the suggestion to carry out studies comparing the social dynamics of men and women living with HIV and how the differences found could be affecting the quality of life and progression of the disease in a different, which would support more objectively the hypotheses that arise about how family dynamics could be influencing the quality of diet and weight gain of HIV+ pregnant women. This could be achieved by including in studies with this type of population, a couples of pregnant women with HIV+, thus observing the complete family dynamics.
