# Supplementary material for: Factors associated with insufficient weight gain among Mexican pregnant women with HIV infection receiving antiretroviral therapy
Source: PLoS One. 2020 May 22;15(5):e0233487. doi: 10.1371/journal.pone.0233487 (PMC7244146; doi:10.1371/journal.pone.0233487)
Supplement: Attachment — Submitted filename: Response to Reviewers 0503.docx [file pone.0233487.s005.docx]

March 5, 2020

Jonathan W. Roginski, Ph.D.

Reviewer 1

PLOS ONE

Dear reviewer:

Thanks, about your suggestions concerning our paper "Factors associated with insufficient weight gain among mexican pregnant women with HIV infection receiving antiretroviral therapy” to your consideration for publication in PLOS ONE.

Now, we respond to each of the observations made by Reviewer 1:

1. Overall: This is an important article and should be published. I suggest the employment of an academic editor, as the English presentation of the work is distracting from the results. Although this study focuses on the 112 women receiving ART, I suggest a similar study be accomplished on the general population of Mexican women, so the results of the women receiving ART be placed in appropriate academic context.

A: The entire manuscript was edited by native speaker in (scientific) American English (certificate annex).

The aim of this study was identified clinical, dietary, and socioeconomic factors associated with insufficient gestational weight gain among Mexican pregnant women with human immunodeficiency virus (HIV) infection; this proposal arises when we found that prevalence of insufficient weight gain among pregnant women with HIV infection is more representative than in women without the disease. (Zonana-Nacach A et al. Salud Pública Mex. 2010; 52 (3):220-225; Perichart-Perera et al. PLoS ONE. 2017; 12(7):e0180874). With this remarkable data, we emphasize the relevance to create new multidisciplinary strategies to pay attention on specific of this population.

We appreciate the suggestion to prepare a comparative study for further work. Such recommendation would help to reinforce whether the factors identified in this study, are specific to this population.

Introduction:

2. “Concerning to a cohort study on HIV seropositive pregnant women who started ART, this analysis found that patients who had a weekly gestational weight gain of less than 100 grams, displayed a higher risk of preterm birth and adverse pregnancy-neonatal outcomes such as low birth weight, concluding that the use of ART is not enough in this kind of population to reduce the risk of these results, because is prevailing a poor nutritional status in spite of ART [12,13].”

--How does this relate to the population of Mexican women that had a weekly gestational weight gain of less than 100 grams and were not HIV seropositive? Answering this question may shift the context of results from the involvement of ART to simply weight gain.

A: About this topic, we know that gestational weight gain below IOM recommendations in pregnant woman without VIH, it has been also associated with adverse pregnancy and neonatal outcomes in other populations (Goldstein RF et al. JAMA. 2017; 317(21):2207-2225) and specifically in Mexican population (Frank R et al. Salud Publica Mex. 2004; 46(1); 23-31;( Samano R et al. PLoS ONE. 2018; 13(7): e0200361), so the ART use cannot be the key answer to the consequences of low weight gain, this was clarified in the manuscript (lines 57-60). Precisely, we want to know if there coexist other factors favoring this insufficient weight gain in pregnant women with HIV and ART use, because there is no information about this in our population. Most of the recent studies that have evaluated the determinants of weight gain in Mexican pregnant women without VIH, have been focused on excessive weight gain (Zonana-Nacach A. Salud Publica Mex. 2010;52 (3);220-225), even assessing how it affects some biochemical markers (Perichart-Perera. PLoS ONE.2017; 12(7):e0180874). This approach is related to the high prevalence of overweight and obesity in Mexican women of reproductive age, as well as, the high prevalence of excessive weight gain that has been reported in studies in the Mexican population (33 al 38%). The population of our study cannot be compared with this generality since as mentioned, it presented a very high proportion of insufficient weight gain (62.5%) and even a significant percentage of weight loss (18.8). This is a result not only of their clinical condition but also of the condition of vulnerability in which they find themselves, as has been raised in the manuscript.

3. The final paragraph seems to indicate a relationship between the receipt of ART and nutritional status. This linkage is unclear, except in that ART is shown to slow the progress of HIV and therefore slow the deterioration of the patient. However, this is different from linking ART to weight gain and improved nutritional status.

A: We agree with your insight stated at the last paragraph of the introduction. We clarified that the use of antiretrovirals is not directly related to nutritional status, but rather, the deterioration is part of the progression of the disease and the presence of poor nutritional status despite the use of antiretrovirals may result from other factors, (lines 60-65).

Results:

4. The statistics stated in the first paragraph are different than those reported in the abstract.

A: The inconsistency, concerning the results in the summary quoted in the first paragraph was corrected.

5. I do not understand the significance of these results: “The percentage of adequacy in energy consumption (r = .22), iron (r = .20), folic acid(r = .20) and zinc (r = .29), as well as the ingesting in grams of protein (r = .29), total fat (r = .20), saturated (r = .28), monounsaturated (r = .26), cholesterol (r = .20) and fiber (r = .20); they correlated with the socioeconomic level (p <0.05).”

A: The correlation of energy consumption and various nutrients with socioeconomic level was considered important to mention, to show how the socioeconomic condition affects the consumption not only of energy but also of nutrients, as a result of consumption patterns of low-income populations (lines 270-273).

6. This statement seems to indicate the authors completed a broader analysis of Mexican women “However, the mainstream (40.2%) showed an insufficient weight gain or weight loss at the time of the evaluation and only 35.7% had a weight gain according to weight gain recommendations [20].” This information must be made more clear.

A: The description of weight gain in the study population was extended, the information was expressly clarified (lines 169-173).

Discussion:

7. The observation surrounding women in unions being at higher risk for insufficient weight gain is important. The authors should expand this and look at other correlative and potentially causal factors.

A: The discussion about women in unions being at higher risk for insufficient weight gain was explained with more detail (lines 232-239).

We agree with the suggestion to carry out studies comparing the social dynamics of men and women living with HIV, and how the difference could be affecting the quality of life and progression of this disease in a different topic, which would support more objectively the hypotheses that arise about how some family dynamics could be influencing the quality of diet and weight gain of HIV+ pregnant women. This could be achieved by including in studies with this type of population, having couples of pregnant women with HIV+, thus observing the complete family dynamics.

8. Based upon the comparison of vitality in Mexican men with HIV compared to that of pregnant Mexican women with ART, it seems like the underlying causal social dynamics could be the subject of an entire other article. It is important and should be publicized.

A: We agree with the suggestion, and indeed we consider it for future studies pondering about masculinities.

March 5, 2020

Diana M. Thomas
Academic Editor
PLOS ONE

Dear academic editor:

Thanks, about your suggestions concerning our paper "Factors associated with insufficient weight gain among mexican pregnant women with HIV infection receiving antiretroviral therapy” to your consideration for publication in PLOS ONE.

Now, we respond to each of the observations made by academic editor:

1. There are many grammatical and typesetting errors.  Many sentences are fragments.  There are acronyms like ART that are not defined. The academic editor suggestions having the paper carefully proofread by a colleague who speaks English as their first language. The academic editor has provided an attachment of handwritten notes, however, these are insufficient edits.  The entire manuscript needs to be edited.

A: The entire manuscript was edited by native speaker in (scientific) American English (certificate annex)

2. Pregnancy weight gain may be incorrect because the study participants may not know their pre-pregnancy weight.  A recent article in the journal Obesity, titled "Do women know their pre-pregnancy weight?" examined women from Mexico and there was evidence that pre-pregnancy weight may have not been known and the self-reported weight deviated from ground truth.  The academic editor suggests using the calculator from this paper to estimate pre-pregnancy weight from the first measured weight as a proxy to check.  Note that because the sample used in that study were healthy pregnancies, it may still be inaccurate.  This is okay, because the authors would have done everything possible to account for this.

A: The pregestational weight was calculated as suggested, according the model to estimate the pregestational weight from the weight in pregnancy that considers height, gestational age, current weight, parity and age using a software (https ://www.pbrc.edu/resea rch-and-facul ty/calculator s/prepregnancy/), reference 24 of the manuscript.
